# Analysis of Inflammatory Features in Suspicious Lesions for Significant Prostate Cancer on Magnetic Resonance Imaging—Are They Mimickers of Prostate Cancer?

**DOI:** 10.3390/cancers17010053

**Published:** 2024-12-27

**Authors:** Juan Morote, Ana Celma, María E. Semidey, Andreu Antolín, Berta Miró, Olga Méndez, Enrique Trilla

**Affiliations:** 1 Department of Urology, Vall d’Hebron Hospital Campus, 08035 Barcelona, Spain; ana.celma@vallhebron.cat (A.C.); enrique.trilla@vallhebron.cat (E.T.); 2 Department of Surgery, Universitat Autònoma de Barcelona, 08193 Bellaterra, Spain; andreuantolin.idi@gencat.cat; 3 Urology Research Group, Vall d’Hebron Research Institute, 08035 Barcelona, Spain; mariaeugenica.semideay@vallhebron.cat (M.E.S.); olga.mendez@vhir.org (O.M.); 4 Department of Pathology, Vall d’Hebron Hospital Campus, 08035 Barcelona, Spain; 5 Department of Radiology, Institut de Diagnòstic per la Imatge, Vall d’Hebron Hospital Campus, 08035 Barcelona, Spain; 6 Statistics Unit, Vall d’Hebron Research Institute, 08035 Barcelona, Spain; berta.miro@vhir.org

**Keywords:** prostate cancer, inflammation, prostatitis, magnetic resonance imaging, prostate biopsy

## Abstract

Background. Inflammatory features can mimic PCa in suspicious MRI-lesions. Objectives: To assess the incidence of inflammatory features in targeted biopsies to suspicious lesions. Methods. A prospective analysis was conducted of 531 MRI-suspicious lesions with Prostate Imaging-Reporting and Data System (PI-RADS) scores of 3 to 5 in 364 men suspected of having PCa. Results. The incidence of inflammatory features in the MRI-suspicious lesions without PCa was 69.6%, compared to 48.1% in those with PCa (*p* < 0.001). Among the suspicious lesions without PCa, the incidence of inflammatory features ranged from 68.6% to 71.2% across the PI-RADS categories (*p* = 0.870). Mild chronic prostatitis increased with higher PI-RADS scores, while acute prostatitis decreased, and granulomatous prostatitis was exclusively observed in patients with PI-RADS scores of 4 and 5. The incidence of inflammatory features in the lesions with insignificant PCa (grade group 1) was 66.7%, compared to 42.7% in those with significant PCa (grade group 2 to 5; *p* = 0.027). The detection of inflammatory features in MRI-suspicious lesions was identified as an independent predictor of a lower likelihood of significant PCa detection, with an odds ratio (OR) of 0.326 (95% CI 0.196–0.541). Mild chronic prostatitis was the only type of prostatitis which was an independent predictor of a lower likelihood of significant PCa, with an OR of 0.398 (95% CI 0.268–0.590). Conclusions. These data suggest that inflammatory features may be considered mimickers of significant PCa on MRI.

## Simple Summary

The early detection of significant prostate cancer (PCa) has markedly improved with the widespread use of pre-biopsy magnetic resonance imaging (MRI) for men suspected of having PCa. However, challenges persist in scenarios involving the low detection rates of significant PCa and the overdetection of insignificant tumors. Inflammatory features are frequently observed in MRI-suspicious lesions that either lack significant PCa or harbor insignificant tumors. These findings suggest that such inflammatory features may mimic significant PCa. Comprehensive radiomic studies focusing on the types and extent of inflammatory features could help reduce the need for unnecessary prostate biopsies.

## 1. Introduction

Prostate cancer (PCa) is the most common malignancy among men worldwide and the third leading cause of cancer-related death [1]. The European Randomized Screening for Prostate Cancer (ERSPC) trial showed a 20% reduction in the PCa-specific mortality among men undergoing screening with serum prostate-specific antigen (PSA) testing and systematic prostate biopsies, compared to those in the control group, after seven years of follow-up [2]. Recently, the Göteborg Randomized Population-Based Prostate Cancer Screening Trial showed a 29% reduction in PCa-specific mortality after 22 years of follow-up [3]. These improvements in PCa-specific mortality were attributed to the increased early detection and treatment of significant PCa, leading to a shift in the focus of population-based screening toward significant cases [4].

The focus on detecting significant PCa during screening was facilitated by the widespread use of prostate magnetic resonance imaging (MRI), now commonly employed to select candidates for prostate biopsy and reducing unnecessary procedures due to its high negative predictive value, reaching up to 97% [5,6,7]. MRI also enables targeted biopsies of lesions suspected of having significant PCa through MRI–transrectal ultrasound (TRUS) fusion imaging technology [8,9]. Therefore, men suspected of having PCa, identified due to a serum PSA elevation, currently undergo an MRI followed by an MRI-TRUS targeted biopsy of lesions with a PI-RADS score of 3 to 5, followed by a systematic prostate biopsy [10]. This new approach avoids unnecessary prostate biopsies and decreases the overdetection of insignificant tumors, although uncertain scenarios remain [11,12,13].

The asymptomatic inflammation of the prostate gland was identified as a frequent non-cancerous cause of serum PSA elevation [14]. In 1999, the National Institute of Health (NIH) classified this non-symptomatic prostatitis as NIH type IV, which is often present when PCa is not detected [15]. Furthermore, several studies suggested asymptomatic prostatitis as a PCa mimicker in MRI [16,17,18]. However, the incidence and type of asymptomatic prostatitis in MRI-suspicious lesions remain poorly analyzed [19].

Our main objective was to analyze the incidence of inflammatory features in targeted biopsies of MRI-suspicious lesions with PI-RADS scores from 3 to 5, focusing our attention on chronic, acute, and granulomatous prostatitis. Our secondary objectives were as follows: (i) to analyze the relationship between inflammatory features found in targeted biopsies without PCa and the PI-RADS score; (ii) to analyze the association of inflammatory features in targeted biopsies with PCa according to the aggressiveness of tumors detected; and (iii) to identify if inflammatory features are independent predictors of significant PCa (grade group 2 or higher).

## 2. Materials and Methods

### 2.1. Design, Setting, and Participants

This is a prospective study analyzing the inflammatory features reported in 531 MRI-suspicious lesions subjected to targeted biopsies in a consecutive series of prostate biopsies carried out in 364 men suspected of having PCa, between 1 January 2022 and 30 September 2023, in one academic center. This study was approved by the ethical committee of the Vall d’Hebron Hospital Campus (PRAG-02/2021).

### 2.2. Diagnostic Approach for Prostate Cancer Detection

Men suspected of having PCa were identified during an opportunistic PCa screening program from a serum prostate-specific antigen (PSA) level above 3.0 ng/mL and/or a suspicious digital rectal examination (DRE). Men referred to our center underwent a 3 Tesla multiparametric MRI in a Magneton Prisma^®^ scanner (Siemens Healthcare, Erlangen, Germany) using a pelvic-phased array coil, following the European Society of Urologic Radiology guidelines recommendations and reported with the Prostate Imaging-Reporting and Data System (PI-RADS) v 2.1 [20,21]. Two- to four-core targeted MRI–transrectal ultrasound fusion imaging biopsies were conducted in men with lesions with PI-RADS scores of 3 to 5, followed by a 12-core systematic biopsy using the Koelis^®^ system (Koelis Advancing PCa, Grenoble, France) via the transperineal route [10]. Biopsy material was sent to the pathology department, where expert pathologists classified the detected tumors according to the International Society of Urologic Pathology grade groups. Significant PCa was identified when the grade group was 2 or higher, and insignificant PCa when the grade group was 1 [22,23]. Inflammatory features were identified in each analyzed core, and prospectively reported as mild, moderate, or severe chronic prostatitis, acute prostatitis, or granulomatous prostatitis [24].

### 2.3. Variables in the Study

The analyzed variables were the age (years), PCa family history (no vs. yes), prostate biopsy type (initial vs. repeated), serum PSA level (ng/mL), DRE (normal vs. suspicious), MRI-prostate volume, and PI-RADS score. Outcome variables were the inflammatory features typified as mild, moderate, and severe chronic prostatitis, acute prostatitis, granulomatous prostatitis, and PCa.

### 2.4. Diagnostic Approach of Inflammatory Features

**Figure 1:**
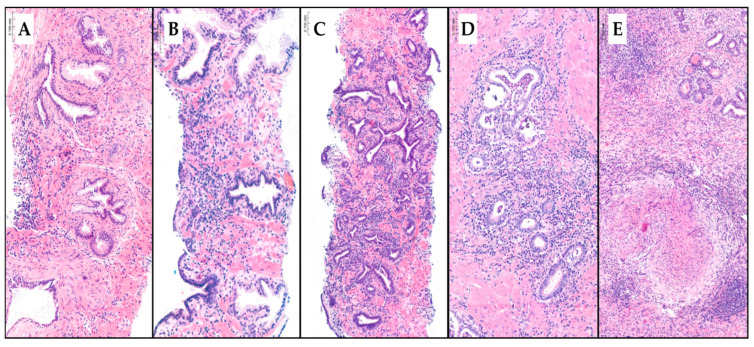
Hematoxylin–eosin-stained slides (0. 100 mm scale) of prostate core biopsy tissue demonstrating mild (**A**), moderate (**B**), and severe chronic prostatitis (**C**), as well as acute prostatitis (**D**) and granulomatous prostatitis (**E**).

Inflammatory features were recorded as mild, moderate, or severe chronic prostatitis; acute prostatitis; or granulomatous prostatitis. The diagnosis was made in hematoxylin and eosin-stained slides. The type of prostatitis depends on the nature of the observed cell infiltration. If the infiltration is composed of lymphocytes and plasma cells, it is classified as chronic prostatitis. The presence of polymorphonuclear neutrophils and eosinophils indicates the acute exacerbation of chronic prostatitis, recorded as acute prostatitis. Granuloma formation is indicative of granulomatous prostatitis. The grading of chronic prostatitis as mild, moderate, or severe is based on inflammatory density. Mild chronic prostatitis typically involves less than 10% of the prostate core volume. Moderate prostatitis affects 10% to more than 50% without confluent inflammatory nests, while severe prostatitis is characterized by more than 50% involvement with confluent inflammatory nests [25,26,27]. When various types of prostatitis coexisted, the most extensive chronic prostatitis was recorded. When the acute exacerbation of chronic prostatitis was observed, acute prostatitis was recorded, as well as granulomatous prostatitis when granuloma formation was observed. Figure 1 represents the type of inflammatory features reported in the hematoxylin and eosin-stained slides.

### 2.5. Statistical Analysis

The quantitative variables were described as medians with interquartile ranges (IQRs), while the qualitative variables were expressed as percentages. The descriptive quantitative variables were compared with the Mann–Whitney U test and the Kruskal–Wallis test results, and the qualitative variables were compared with chi-square test results. Logistic regression analysis was used to analyze the inflammatory findings as predictors of significant PCa. Odds ratios (ORs) and 95% confidence intervals (CIs) were calculated in a univariate and multivariate analysis. Statistical significance was allowed when the *p*-value was lower than 0.05. Statistical analyses were computed using SPSS^®^ v.29 (IBM, statistical package for social sciences, San Francisco, CA, USA).

## 3. Results

### 3.1. Characteristics of Study Population and Guided-Biopsy Lesions

The overall characteristics of the study population are summarized in Table 1. We note that, among the 364 men suspected of having PCa due to a serum PSA level above 3.0 ng/mL and/or a suspicious DRE, the median age was 68 years, the median serum PSA level was 6.1 ng/mL, and the percentage of suspicious DREs was 20.1%. A total of 25 men (6.9%) had an existing PCa family history, and 41 (11.2%) had previous negative prostate biopsies. A total of 196 men carried one suspicious lesion, while 142 (51.7%) presented two and 26 (7.1%) had three suspicious lesions. The percentage of index lesions with a PI-RADS score of 3 was 36.0%, whereas those with lesions with PI-RADS scores of 4 and 5 made up proportions of 42.6% and 21.4%, respectively. The overall detection rate of PCa was 46.1%, with 35.7% being significant PCa and 10.4% being insignificant PCa.

The number of targeted biopsied suspicious lesions was 531, with a median size of 10 mm. A total of 440 (82.9%) suspicious lesions were located in the peripheral zone, 20 (3.8%) were located in the central or transitional zone, and 13.4% were located in the anterior zone. The percentage of lesions with a PI-RADS score of 3 was 48.2%, while 36.2% and 15.6% had PI-RADS scores of 4 and 5, respectively. The overall PCa detection rate per lesion was 29.4%, with 20.3% being significant PCa and 8.5% being insignificant PCa, as shown in Table 2.

### 3.2. Distribution of Inflammatory Features in Suspicious Lesions Without and with Prostate Cancer

**Figure 2:**
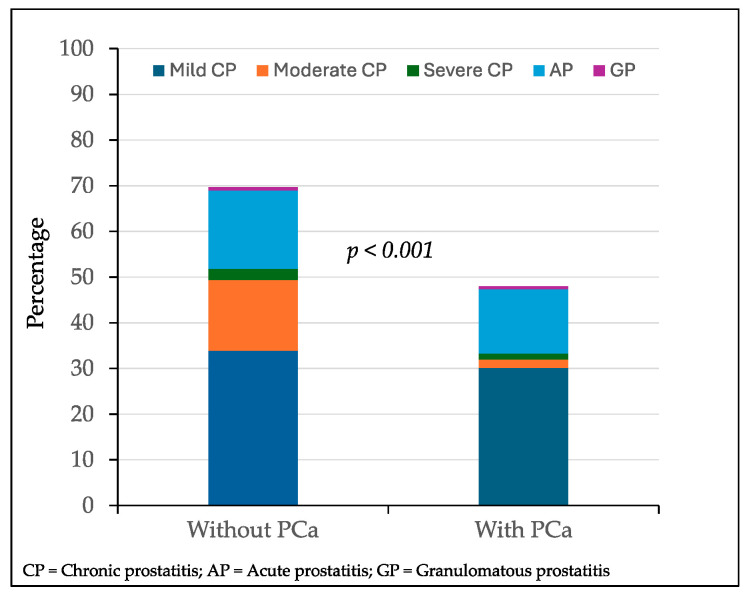
Distribution of inflammatory findings in targeted biopsies of suspicious lesions based on the presence of prostate cancer.

Overall inflammatory features were found in 336 of 531 MRI-suspicious lesions (63.3%). Mild chronic prostatitis was detected in 174 lesions (33.4%), followed by acute prostatitis in 86 lesions (16.5%), moderate chronic prostatitis in 61 (11.7%), severe chronic prostatitis in 11 (2.1%), and granulomatous prostatitis in 4 lesions (0.7%). Overall inflammatory features were found in 261 of 375 lesions with PCa (69.6%) and 75 of 156 with PCa (48.1%), *p* < 0.001. The cases of mild, moderate, or severe chronic prostatitis, acute prostatitis, and granulomatous prostatitis among the 375 targeted biopsied lesions without PCa and 156 lesions with PCa are represented in Figure 2. Mild chronic prostatitis was detected in 127 lesions (33.9%) without PCa, while it was detected in 47 (30.1%) with PCa (*p* = 0.128). Moderate chronic prostatitis was observed in 58 lesions (15.5%) without PCa, while it was found in 3 (1.9%) with PCa (*p* < 0.001); severe chronic prostatitis was found in nine (2.4%) and two lesions (1.3%), respectively (*p* = 0.089). Acute prostatitis was detected in 64 lesions (12.0%) without PCa, while it was found in 22 (14.1%) with PCa (*p* = 0.113). Granulomatous prostatitis was detected in three lesions without PCa (0.1%) and one lesion (0.6%) with PCa (*p* = 0.568).

### 3.3. Distribution of Inflammatory Features in Targeted Biopsies According to the PI-RADS Category

**Figure 3:**
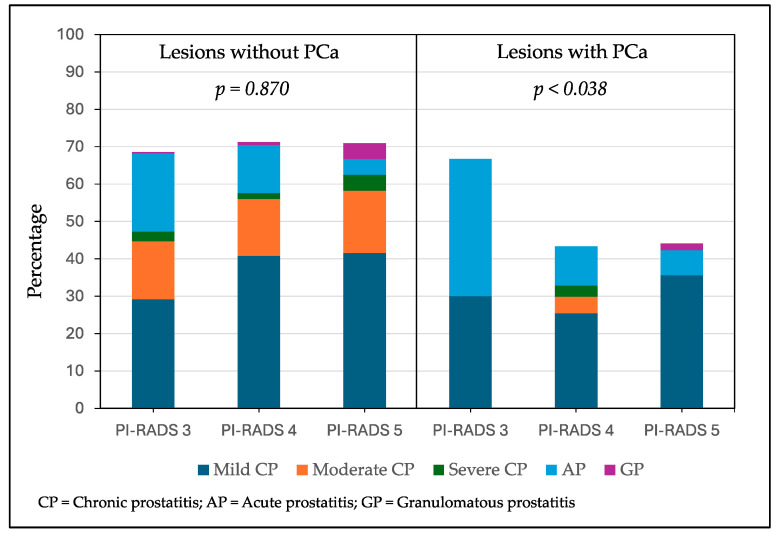
Distribution of inflammatory findings in targeted biopsy lesions without and with prostate cancer, stratified by PI-RADS score.

The distribution of mild, moderate, and severe chronic prostatitis, acute prostatitis, and granulomatous prostatitis according to the PI-RADS score of the biopsied lesions without PCa is represented in Figure 3. There were 66 lesions (29.2%) with mild chronic prostatitis of a PI-RADS score of 3, while 51 (40.8%) lesions had a PI-RADS score of 4 and 10 (41.6%) lesions had a PI-RADS score of 5 (*p* = 0.014). Moderate chronic prostatitis was detected in 35 (15.5%) lesions with a PI-RADS score of 3, 19 (15.2%) lesions with a PI-RADS score of 4, and 4 (16.6%) lesions with a PI-RADS score of 5 (*p* = 0.367). Severe chronic prostatitis was found in six lesions (2.6%) with a PI-RADS score of 3, two (1.6%) with a PI-RADS score of 4, and one (4.3%) with a PI-RADS score of 5 (*p* = 0.536). Acute prostatitis was observed in 47 lesions (20.8%) with a PI-RADS score of 3, 16 (12.8%) with a PI-RADS score of 4, and 1 (2.9%) with a PI-RADS score of 5 (*p* = 0.006). Granulomatous prostatitis was found in one lesion (0.4%) with a PI-RADS score of 3, one (0.8%) with a PI-RADS score of 4, and one (2.9%) with a PI-RADS score of 5 (*p* = 0.024). Overall inflammatory features were reported in 155 (68.6%) out of 226 lesions with a PI-RADS score of 3, 89 (71.2%) out of 125 lesions with a PI-RADS score of 4, and 17 (70.8%) out of 24 lesions with a PI-RADS score of 5 (*p* = 0.870).

The distribution of mild, moderate, and severe chronic prostatitis, acute prostatitis, and granulomatous prostatitis according to the PI-RADS score of the biopsied lesions with PCa is also represented in Figure 3. There were 9 lesions (30.0%) with mild chronic prostatitis of a PI-RADS score of 3, while 17 (25.4%) in PI-RADS score of 4, and 21 (35.6%) with PI-RADS score of 5. Moderate chronic prostatitis was not found in lesions with a PI-RADS score of 3, three (4.5%) in lesions with a Pi-RADS score of 4, and none in lesions with a PI-RADS score of 5 (*p* = 0.438). Severe chronic prostatitis was not found in lesions with a PI-RADS score of 3, two (3.0%) with a PI-RADS score lesions of 4, and none with a PI-RADS score of 5 (*p* N.A.). Acute prostatitis was observed in 11 (36.7%) lesions with a PI-RADS score of 3, seven (10.4%) with a PI-RADS score of 4, and four (6.8%) with a PI-RADS score of 5 (*p* = 0.037). Granulomatous prostatitis was only found in one lesion (1.7%) with a PI-RADS score of 5 (*p* N.A.). Overall inflammatory features were reported in 19 (63.3%) out of 30 lesions with a PI-RADS score of 3, 29 (43.2%) out of 67 lesions with a PI-RADS score of 4, and 25 (42.4%) out of 59 lesions with a PI-RADS score of 5 (*p* = 0.021).

### 3.4. Distribution of Inflammatory Findings in Targeted Biopsies of Suspicious Lesions with Prostate Cancer According to Its Aggressiveness

**Figure 4:**
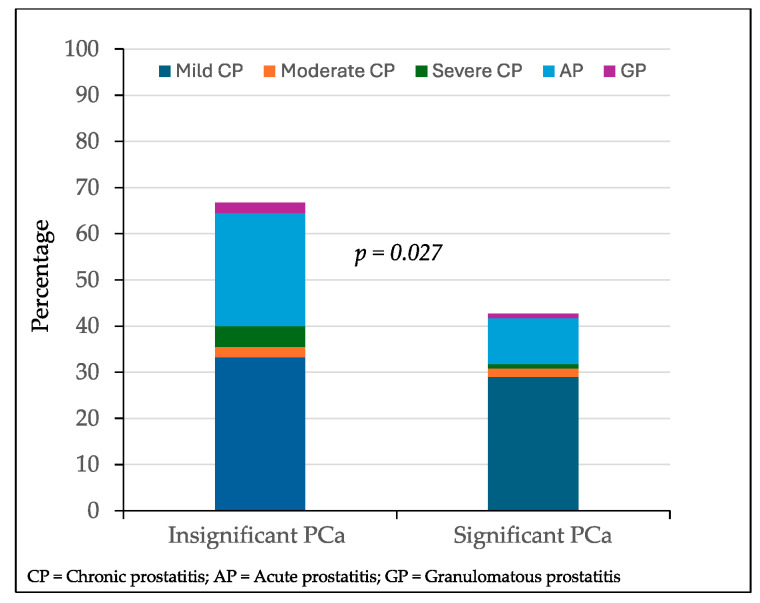
Distribution of inflammatory findings in targeted biopsy lesions with prostate according, categorized by tumor aggressiveness.

Among 156 targeted biopsied lesions with PCa, inflammatory symptoms were observed in 30 of 45 lesions (66.7%) with insignificant PCa compared to 47 of 110 lesions (42.7%) with significant PCa (*p* = 0.027). The distribution of the prostatitis type according to the detection of insignificant or significant PCa is represented in Figure 4. Mild chronic prostatitis was detected in 15 lesions with insignificant PCa (33.3%) and in 32 lesions (29.1%) with significant PCa (*p* = 0. 453). Moderate chronic prostatitis was detected in one lesion with insignificant PCa (2.2%) and in two (1.8%) with significant PCa (*p* = 0.673). Severe chronic prostatitis was detected in two lesions with insignificant PCa (2.2%) and in one with significant PCa (*p* = 0.876). Acute prostatitis was detected in 11 lesions with insignificant PCa (25.5%) and in 11 (10.0%) with significant PCa (*p* = 0.036). Granulomatous prostatitis was detected in one lesion (*p* = 1.000).

### 3.5. Analysis of Associations with Significant Prostate Cancer and Its Prediction of Characteristics of Suspicious Lesions and Reported Inflammatory Findings

The results of a univariate and multivariate analysis of the characteristics of the suspicious targeted biopsied lesions, the reported overall inflammation, and, specifically, the detection of chronic, acute, and granulomatous prostatitis, describing their association with and prediction of significant PCa, are summarized in Table 3.

We noted in the univariate analysis that the size of the lesion, the PI-RADS score, the detection of inflammatory lesions, and, specifically, chronic prostatitis was associated with the detection of significant PCa in those lesions. However, the location of these lesions (peripheral, central/transition, and anterior) and the detection of acute prostatitis and granulomatous prostatitis were not associated with the detection of significant PCa. The multivariate analysis shows that the lesion size, their location in the prostate gland, and, specifically, the detection of acute prostatitis and granulomatous prostatitis did not predict the detection of significant PCa. On the contrary, the increase in the PI-RADS score of the suspicious lesions increased the risk of significant PCa, while the presence of inflammatory findings, and specifically of chronic prostatitis, decreased the risk of significant PCa.

## 4. Discussion

The present study demonstrated that inflammatory features are frequently detected in targeted biopsies of MRI-suspicious lesions without PCa, consistent with findings previously reported in systematic biopsies [28,29]. In the pre-MRI era, Schatterman et al. observed that inflammation was present in nearly all systematic biopsies after excluding pre-malignant lesions and symptomatic prostatitis. They also noted that increases in the serum PSA level and PSA density were usually typically associated with epithelial disruption caused by the aggressiveness of inflammatory infiltrates rather than their extent [29]. Our findings revealed that prostatitis features in the targeted biopsies of lesions without PCa were more common than those observed in targeted biopsies where PCa was present. The overall percentage of inflammatory features across the targeted lesions of various PI-RADS scores without PCa remained stable, in contrast with the decrease reported in other studies [30]. However, we observed an increased prevalence of mild chronic prostatitis in lesions with PI-RADS scores of 4 and 5 compared to those with a PI-RADS score of 3. The incidence of intermediate and severe chronic prostatitis was consistent across the PI-RADS categories, while the incidence of acute prostatitis decreased with higher PI-RADS scores. Granulomatous prostatitis, an uncommon inflammatory condition, was exclusively observed in lesions with PI-RADS scores of 4 and 5. Previous studies suggested that granulomatous prostatitis tends to appear in the lesions with the highest PI-RADS scores, with some exploring radiomic features to differentiate it from significant PCa [18,31,32,33,34,35]. In our study, we observed a significantly increased incidence of mild chronic prostatitis and acute prostatitis in lesions with insignificant PCa compared to those with significant PCa. Notably, granulomatous prostatitis was observed in the lesions with significant and insignificant PCa [31,32,33]. Regarding the inflammatory features in lesions containing PCa, we observed a distribution different from that in lesions without PCa. Specifically, there was a higher incidence of acute prostatitis, particularly in lesions classified as having a PI-RADS score of 3.

The incidence of inflammatory features was lower in the targeted biopsies of lesions with significant PCa compared to those with insignificant PCa. In this study, we observed that detection of inflammation in the targeted biopsies of MRI-suspicious lesions was an independent predictor of a lower likelihood of detecting significant PCa. Consistent with our findings, previous studies reported that both acute prostatitis [36,37] and chronic prostatitis [36,38,39,40,41,42] are associated with a reduced risk of future PCa.

The high percentage of inflammatory features in negative targeted biopsies of suspicious lesions suggest that these features can mimic PCa in MRI findings. Although our study was not specifically designed to confirm this hypothesis, the high incidence of inflammatory features in suspicious lesions without PCa supports this possibility.

In 2016, Jyoti et al. conducted a retrospective analysis of 228 MRI-suspicious lesions in 137 men suspected of having PCa. PCa was detected in 55 lesions (24.1%), while inflammatory features were identified in 62 lesions (27.2%) without PCa, with incidences of 58% with a PI-RADS score of 3, 39% with a PI-RADS score of 4, and 3% with a PI-RADS score of 5. Their study suggested that the prevalence of inflammatory features decreases with higher PI-RADS scores [43]. In contrast, we showed a higher incidence of inflammatory features, at approximately 70% across all the PI-RADS categories. Notably, we observed an increased prevalence of mild chronic prostatitis with PI-RADS scores of 4 and 5 compared to lesions with a PI-RADS score of 3. Conversely, the incidence of acute prostatitis decreased progressively with higher PI-RADS scores, and granulomatous prostatitis emerged as a rare finding in lesions with PI-RADS scores of 4 and 5. These discrepancies may reflect differences in the radiomic characteristics of various types of prostatitis. Jyoti et al.’s study did not specify the types of inflammatory lesions identified, reporting a high median prostate volume and analyzing the lesions irrespective of the presence or absence of PCa. Some authors specifically highlighted granulomatous prostatitis as a major mimicker of PCa, particularly in lesions with the highest PI-RADS scores [18,31,32,33,34,35].

In 2018, Gordestsky et al. analyzed the incidence of benign findings in 41 retrospectively selected cases with prior negative biopsies, examining 62 MRI-suspicious lesions sampled through targeted biopsies and additional systematic biopsies. The authors reported inflammatory features in 29% of the targeted biopsies and 14.6% of the systematic biopsies. Chronic prostatitis was identified in 10% of the targeted biopsies and 7.1% of the systematic biopsies, while granulomatous prostatitis was detected in 3.2% and 2.5%, respectively [44]. Although our study did not compare the incidence of inflammatory features between targeted and systematic biopsies, we did assess the incidence of inflammatory features in targeted biopsies according to the presence of PCa. The incidence of inflammatory features was higher in lesions without PCa compared to those with PCa, and that in significant PCa lesions was lower than in those with insignificant PCa.

In 2019, Rourke at al. retrospectively identified 43 men who consecutively underwent biopsies for 61 suspicious lesions observed on pre-biopsy prostate MRIs. These men received both targeted and systematic biopsies. Pathologists subsequently reviewed the pathology, identifying inflammation in 43 lesions (70.5%). Inflammatory features were found in 54.5% of the targeted biopsies without PCa, compared to 40% of the cases where PCa was detected only through systematic biopsies. While they reported a similar incidence of inflammatory features, we observed a lower incidence in the suspicious lesions with PCa. However, no association was found between inflammatory lesions and the PI-RADS scores or the aggressiveness of the PCa, consistent with the findings of our study [19].

In 2020, Pepe et al. analyzed the detection of granulomatous prostatitis in 105 men with lesions of a PI-RADS score of 5 who underwent targeted prostate biopsies. PCa was detected in 91 cases (86.7%), with significant PCa identified in 89 cases (84.5%). Among 16 men without significant PCa, granulomatous prostatitis was detected in 6 (37.7%). The authors reported that these men underwent specific antibiotic treatment, after which none required repeated biopsies. All the patients demonstrated their serum PSA level and PSA density, along with a reduction in their PI-RADS score to 3 or lower [33]. Bertelli et al. recently analyzed 11 cases of granulomatous prostatitis, including 4 cases with lesions of PI-RADS score of 4 and 6 with lesions of PI-RADS score of 5, as reported using PI-RADS version 2.1. Among them, seven cases involved nonspecific lesions, while five men had previously received Bacillus Calmette–Guérin vesical instillations to prevent recurrences of non-muscle-invasive bladder cancer. All the lesions exhibited a low signal intensity on T2-weighted images, restricted diffusion with hyperintensity in diffusion-weighted imaging, and low apparent diffusion coefficient values. With dynamic contrast-enhanced imaging, most cases of specific granulomatous prostatitis demonstrated high peaks and persistent enhancement. Additionally, most of these latter lesions showed an avascular core and peripheral rim enhancement. This study reinforced the association between granulomatous prostatitis and high-PI-RADS-score lesions, consistent with our findings, and suggested the potential role of radiomic features for differentiating these lesions from those of significant PCa [18]. Recent studies also highlighted promising functional imaging techniques for differentiating chronic prostatitis from significant PCa, such as quantitative contrast-enhanced perfusion kinetics on multiparametric MRI [17,29,45,46].

One of the strengths of the current study is that a large, prospective, and correlative cohort of cases was analyzed, focusing on the type and extent of prostatitis observed in targeted biopsies. However, several limitations should be noted. First, the analysis was restricted to asymptomatic prostatitis, excluding other benign or pre-malignant conditions that may also mimic PCa. Second, this study was not designed to identify radiomic characteristics capable of differentiating PCa from inflammatory features. Furthermore, the diversity and extent of inflammatory features complicate the interpretation of the findings. Lastly, while targeted core biopsies provide valuable insights, they do not fully represent the entire tissue of suspicious lesions nor do prostate biopsies capture the pathology of the whole prostate gland.

Future well-designed radiomic studies focusing on the differentiation of specific inflammatory features and their extent in MRI-suspicious lesions could provide valuable insights into identifying true mimickers of significant PCa [47]. New biomarkers will be recommended for future development to complement MRI results, enabling the distinction between PCa from benign inflammatory features, thereby reducing the need for unnecessary biopsies [48,49,50,51]. Once the diagnosis of inflammatory features is made, it may be advisable to adopt new anti-inflammatory lifestyle habits such us engaging in physical activity, quitting smoking, reducing alcohol intake, and following anti-inflammatory drug to reduce their chronic inflammation, potentially decreasing the future risk of PCa [52,53,54,55,56,57,58,59]. Such advancements could help reduce unnecessary prostate biopsies, particularly in uncertain scenarios characterized by low detection rates of significant PCa and the persistent overdetection of insignificant PCa [47].

## 5. Conclusions

Asymptomatic inflammatory features were observed in approximately 65% of the MRI-suspicious lesions, occurring more frequently in lesions with PCa than in those without. The most common inflammatory finding was mild chronic prostatitis, followed by acute prostatitis, moderate chronic prostatitis, severe chronic prostatitis, and granulomatous prostatitis. Granulomatous prostatitis was rare and primarily found in lesions with PI-RADS scores of 4 and 5. The inflammatory findings remained consistent across the PI-RADS categories in lesions without PCa. However, their incidence was significantly higher in lesions without PCa compared to those with PCa, as well as in lesions with insignificant PCa compared to those with significant PCa. Overall inflammatory features were identified as an independent predictor of a lower likelihood of detection of significant PCa. These findings suggested that overall inflammatory features could serve as potential mimickers of significant PCa.

## Figures and Tables

**Table 1:** Characteristics of study population.

Characteristic	Measurement
Number of cases	364
Median age, years (IQR)	68 (62–74)
PCa family history, *n* (%)	25 (4.7)
Previous negative biopsy, *n* (%)	41 (11.2)
Median serum PSA, ng/mL (IQR)	6.1 (4.3–9.6)
Suspicious DRE, *n* (%)	75 (20.1)
Median prostate volume, mL (IQR)	53 (39–71)
Number of MRI suspicious lesions per case, *n* (%)	
1	364 (100)
2	142 (51.7)
3	25 (6.9)
PI-RADS score of index lesion, *n* (%)	
3	131 (36.0)
4	155 (42.6)
5	78 (21.4)
Overall PCa detection, *n* (%)	168 (46.1)
Significant PCa detection, *n* (%)	130 (35.7)
Insignificant PCa detection, *n* (%)	38 (10.4)

IQR: interquartile range; PSA: prostate-specific antigen; DRE: digital rectal examination; PI-RADS: Prostate Imaging-Reporting and Data System; PCa: prostate cancer.

**Table 2:** Characteristics of MRI-suspicious lesions that underwent targeted biopsy.

Characteristic	Measurement
Number of suspicious lesions, *n* (%)	531
Median size, mm (IQR)	10 (8–15)
Localization	
Peripheral zone, *n* (%)	440 (82.9)
Central/Transition zone, *n* (%)	20 (3.8)
Anterior zone, *n* (%)	71 (13.4)
PI-RADS score	
3, *n* (%)	256 (48.2)
4, *n* (%)	192 (36.2)
5, *n* (%)	83 (15.6)
Suspicious lesions without PCa, *n* (%)	375 (70.6)
Suspicious lesions with PCa, *n* (%)	156 (29.4)
With significant PCa, *n* (%)	110 (20.3)
With insignificant PCa, *n* (%)	45 (8.5)

IQR: interquartile range; PI-RADS: Prostate Imaging-Reporting and Data System; PCa: prostate cancer.

**Table 3:** Univariate and multivariate analyses of the characteristics of targeted biopsy suspicious lesions and inflammatory finding for predicting significant prostate cancer.

Characteristic	Univariate Analysis		Multivariate Analysis	
	Odds Ratio (95% CI)	*p* Value	Odds Ratio (95% CI)	*p* Value
Size, Ref. one mm	1.100 (1.018–1.142)	<0.001	0.988 (0.939–1.040)	0.646
Localization zone, Ref. peripheral	1.108 (0.827–1.483)	0.493	1.109 (0.783–1.569)	0.561
PI-RADS score, Ref. 3 score	6.168 (4.295–8–858)	<0.001	6.466 (4.176–10.013)	<0.001
Inflammatory lesions, Ref. yes	0.325 (0.211–0.500)	<0.001	0.326 (0.196–0.541)	<0.001
Chronic prostatitis. Ref. yes	0.486 (0.351–0.674)	<0.001	0.398 (0.268–0.590)	<0.001
Acute prostatitis. Ref. yes	0.565 (0.295–1.081)	0.085	1.046 (0.488–2.342)	0.907
Granulomatous prostatitis, Ref. yes	1.278 (0.132–12.410)	0.832	0.551 (0.038–7.903)	0.661

PI-RADS: Prostate Imaging-Reporting and Data System; CI: confidence interval.

## Data Availability

Data are available upon request to the corresponding author.
